# Vascular tumor or malformation of the pediatric airway?

**DOI:** 10.1002/ccr3.2567

**Published:** 2019-12-03

**Authors:** Joana Pires, Mafalda Ferreira, João Elói Moura, Felisberto Maricato

**Affiliations:** ^1^ Department of Otolaryngology Coimbra Hospital and University Centre Coimbra Portugal

**Keywords:** airway management, endoscopic surgical procedure, pediatrics, vascular malformations

## Abstract

Distinguishing airway vascular tumors from malformations can be challenging. Failure to treat a suspected airway hemangioma with a beta‐blocker and systemic corticosteroid should raise suspicion of a possible airway malformation.

## Graphical abstract

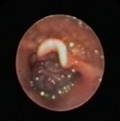

Vascular anomalies of the pediatric airway are classified as tumors, commonly hemangiomas or malformations, based on clinical and histologic characteristics. We describe a case report of a 3‐year‐old male patient presenting with obstructive sleep apnea. Airway endoscopy detected a supraglottic large, violaceous, heterogeneous mass, with insertion in the right aryepiglottic fold and posterior wall of the hypopharynx, mobile with breathing, only allowing visualization of the anterior and middle portions of the vocal folds (Figure 1). A computed tomography angiography described an expansive lesion with soft tissue density, causing a reduction of about 60% of the laryngeal lumen, with a contrast enhancement effect in its central portion, which was suggestive of a hemangioma (Figure 2). Treatment with oral propranolol (2 mg/kg/day) and intravenous prednisolone 1 mg/kg (with weaning) was initiated, but after 6 months, there was no clinical or endoscopic improvement, so we decided to perform endoscopic surgical excision (Figure 3). The histologic study was compatible with a venous malformation. There was an immediate clinical improvement and at 1 year of follow‐up, there were no endoscopic signs of recurrence. Distinguishing airway vascular tumors from malformations can be challenging. Failure to treat a suspected airway hemangioma with a beta‐blocker and systemic corticosteroid should raise suspicion of a possible airway malformation.

**Figure 1:**
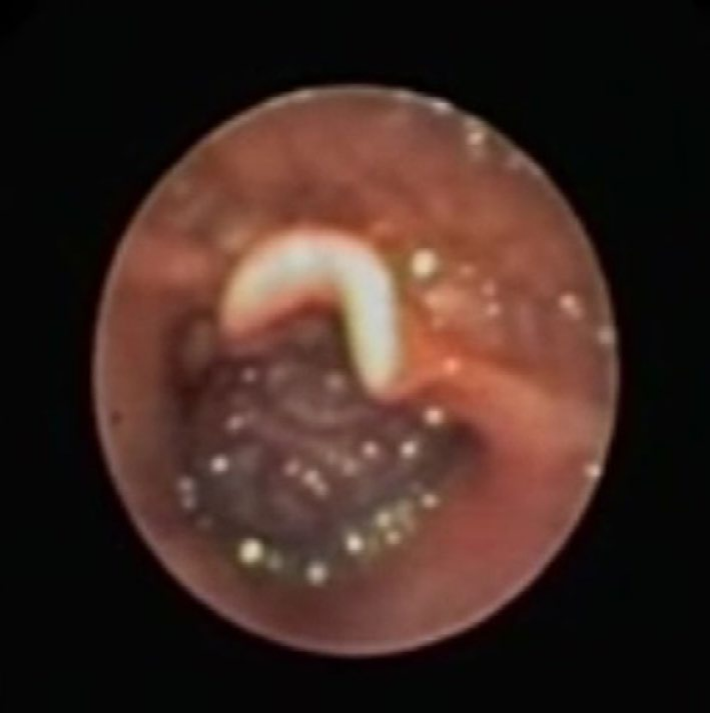
Supraglottic mass with insertion in the right aryepiglottic fold and posterior wall of the hypopharynx

**Figure 2:**
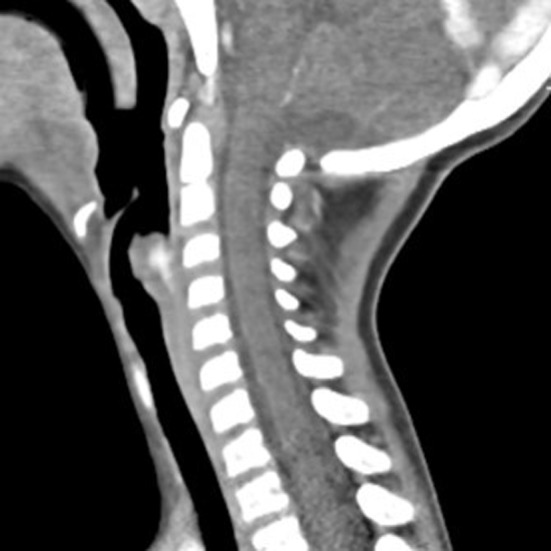
Computed tomography angiography showing an expansive lesion with soft tissue density, causing a reduction of about 60% of the laryngeal lumen, with a contrast enhancement effect in its central portion

**Figure 3:**
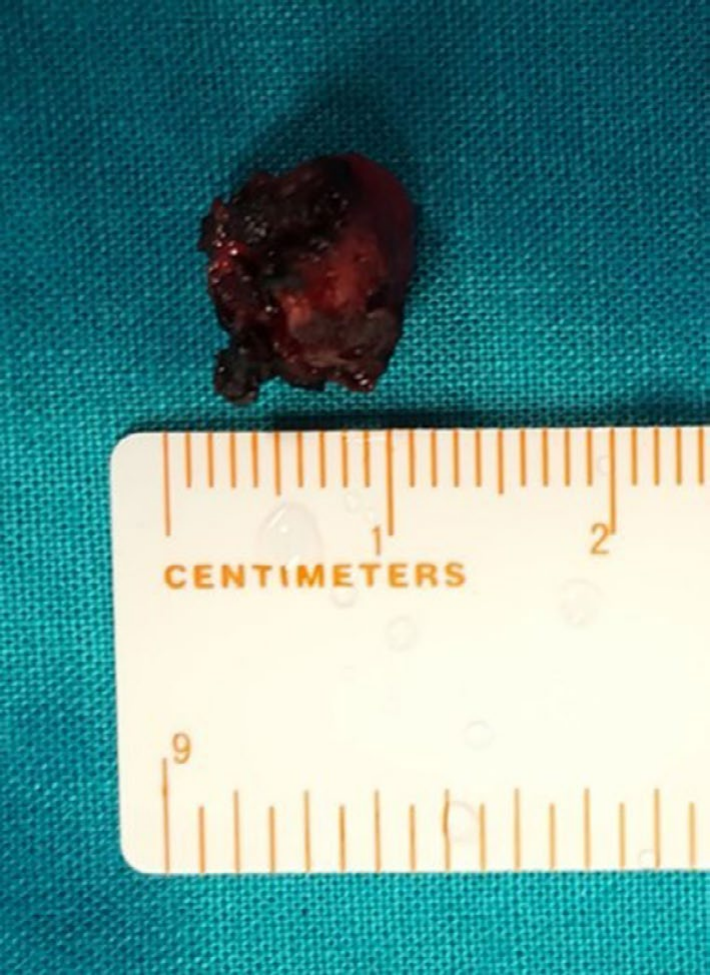
Venous malformation after surgical excision

## CONFLICT OF INTEREST

None declared.

## AUTHOR CONTRIBUTIONS

Joana Pires: revised the literature and drafted the work. Mafalda Ferreira: revised the literature. João Elói Moura: Assistant surgeon. Involved in acquisition of clinical data and revised the work. Felisberto Maricato: Main surgeon. Revised the work and gave final approval of the version to be published.

